# ﻿A new species and two new records of *Indomasonaphis* Verma, 1971 (Hemiptera, Aphididae) from China

**DOI:** 10.3897/zookeys.1253.157130

**Published:** 2025-09-29

**Authors:** Shokhruz Nazarov, Ying Xu, Li-Yun Jiang, Ge-Xia Qiao

**Affiliations:** 1 State Key Laboratory of Animal Biodiversity Conservation and Integrated Pest Management, No. 1-5 Beichen West Road, Chaoyang District, Beijing 100101, China State Key Laboratory of Animal Biodiversity Conservation and Integrated Pest Management Beijing China; 2 Institute of Zoology, Academy of Sciences of the Republic of Uzbekistan, Tashkent, Uzbekistan University of Chinese Academy of Sciences Beijing China; 3 College of Life Science, University of Chinese Academy of Sciences, Beijing, China Institute of Zoology, Academy of Sciences of the Republic of Uzbekistan Tashkent Uzbekistan; 4 College of Life Science, Ludong University, Yantai, Shandong, China Ludong University Yantai China

**Keywords:** Aphidinae, clavate siphunculi, DNA barcode, key, new record, new species, taxonomy

## Abstract

*Indomasonaphis* Verma, 1971 is a small genus primarily distributed in the southern Himalayan region. The genus is morphologically characterized by a concave frons; sparse, long, thick, and blunt or capitate dorsal setae; long clavate siphunculi; spinulose tarsi; and conspicuously hairy cauda. Based on the examination of Chinese specimens, the genus *Indomasonaphis* is recorded for the first time in China. A new species, *Indomasonaphis polygoni* Qiao & Xu, **sp. nov.** on *Polygonum* sp. is described here. Additionally, *Indomasonaphis anaphalidis* (Basu, 1964) and *Indomasonaphis rumicis* (Chakrabarti & Raychaudhuri, 1975) are newly recorded here from China. DNA barcode sequences of two species in the genus, *I. anaphalidis* (Basu) and *I. polygoni* Qiao & Xu, **sp. nov.** were obtained for the first time. Keys to the Chinese species in this genus have been constructed.

## ﻿Introduction

*Indomasonaphis* Verma, 1971 is a small genus within the tribe Macrosiphini (Hemiptera, Aphidinae), with five species currently recorded worldwide (Favret 2025). The genus was established by Verma (1971), who designated *Indomasonaphis indicum* Verma, 1971 as the type species. The main morphological characteristics of the genus are as follows: frons concave, with well-developed and diverging antennal tubercles; dorsal setae sparse, long, thick, and blunt or capitate; siphunculi long clavate; tarsi with spinulose imbrications; and cauda with numerous setae. *Indomasonaphis anaphalidis* (Basu, 1964), originally described as *Masonaphis anaphalidis*, feeds on *Anaphalis triplinervis* (Asteraceae) in India (Basu 1964), and was later transferred to the genus *Indomasonaphis* (Remaudière and Remaudière 1997). *Indomasonaphis indicum* was subsequently regarded as a junior synonym of *Indomasonaphis anaphalidis* (Eastop and Blackman 2005). Later, three species in India were described as follows: *Masonaphis inulae* Ghosh & Raychaudhuri, 1972 on *Inula cappa* (Asteraceae) and *Rhododendron* sp. (Ericaceae); *Masonaphis rumicis* Chakrabarti & Raychaudhuri, 1975 on *Rumex* sp. (Polygonaceae); and *Indumasonaphis chakrabartii* Bhattacharya, 1991 on *Inula* sp. (Asteraceae). Recent taxonomic revisions have reclassified *Masonaphis inulae*, *Masonaphis rumicis*, and *Indumasonaphis chakrabartii* under the genus *Indomasonaphis* (Favret 2025). Das and Ghosh (2002) described *Neomasonaphis vasesiphon* infesting *Senecio* sp. from Binsar, Uttaranchal, India. Chakrabarti (2024) included this species under the genus *Indomasonaphis* and suspected that it might be a junior synonym of *Indomasonaphis anaphalidis*. After having examined the Chinese specimens in the genus *Indomasonaphis*, one new species and two newly recorded species were identified and are described here.

## ﻿Materials and methods

Aphid terminology used in this paper follows that of Verma (1971), and Favret and Aphid Taxon Community (2025), except that body length is measured from the frons to the end of the cauda. The specimens were examined using a Leica DM 2500 compound microscope and photographed with a Leica MC 5400 camera. The unit of measurement is millimeters (mm). All morphometric data are listed in Table 1.

**Table 1.** Morphometric data on species of *Indomasonaphis* in China based on examined specimens. All measurements are in mm.

Parts		Indomasonaphis anaphalidis	Indomasonaphis polygoni Qiao & Xu, sp. nov.	Indomasonaphis rumicis
		Apterous viviparous females (n = 2)	Apterous viviparous females (n = 10)	Apterous viviparous females (n = 2)
Length (mm)	Body length	4.419–4.450	5.147–6.367	3.375–3.956
	Body width	1.847–1.966	1.931–2.66	1.708–2.068
	Antennae	6.125–6.677	3.899–5.891	4.764
	Ant. I	0.311–0.324	0.257–0.328	0.256–0.282
	Ant. II	0.152–0.153	0.150–0.182	0.138–0.145
	Ant. III	1.393–1.567	1.099–1.469	1.104–1.127
	Ant. IV	1.144–1.343	0.891–1.143	0.851–0.896
	Ant. V	1.004–1.107	0.718–0.927	0.652–0.704
	Ant. VIb	0.331–0.345	0.315–0.376	0.193–0.200
	PT	1.776–1.852	1.483–1.519	1.436
	URS	0.218–0.241	0.185–0.209	0.153–0.178
	Hind femur	1.914–2.132	1.970–2.247	1.257–1.514
	Hind tibia	3.689–3.962	3.375–3.812	2.671–2.853
	2HT	0.191–0.192	0.195–0.224	0.173–0.173
	SIPH	1.095–1.252	0.642–0.793	0.605–0.672
	BW SIPH	0.124–0.127	0.102–0.166	0.078–0.086
	SW SIPH	0.176–0.216	0.099–0.127	0.125–0.157
	DW SIPH	0.080–0.086	0.085–0.100	0.065–0.077
	Cauda	0.557–0.628	0.474–0.583	0.331–0.343
	BW cauda	0.178–0.217	0.209–0.314	0.191–0.198
	Ant. III BD	0.052–0.055	0.051–0.063	0.044–0.045
	MW hind tibia	0.062–0.067	0.075–0.098	0.061–0.072
	Cephalic setae	0.083–0.105	0.090–0.121	0.054–0.072
	Setae on tergite I	0.082–0.114	0.089–0.127	0.034–0.035
	Setae on tergite VIII	0.142–0.143	0.092–0.143	0.060–0.064
	Setae on Ant. III	0.042–0.043	0.048–0.071	0.019–0.029
	Setae on hind tibia	0.074–0.077	0.088–0.134	0.057–0.076
Ratio	Body length / Body width	2.25–2.41	2.26–2.67	1.91–1.98
	Whole antennae / Body	1.39–1.50	0.66–0.99	1.20
	Hind femur / Ant. III	1.36–1.37	1.34–1.84	1.12–1.37
	Hind tibia / Body	0.84–0.89	0.60–0.69	0.68–0.85
	Ant. I / Ant. III	0.20–0.23	0.20–0.26	0.23–0.26
	Ant. II / Ant. III	0.10–0.11	0.11–0.15	0.12–0.13
	Ant. IV / Ant. III	0.82–0.86	0.73–0.85	0.77–0.80
	Ant. V / Ant. III	0.71–0.72	0.56–0.69	0.59–0.63
	Ant. VIb / Ant. III	0.22–0.24	0.22–0.32	0.17–0.18
	PT / Ant. III	1.18–1.28	1.04–1.38	1.27
	PT / Ant. VIb	5.36–5.37	3.96–4.71	7.18
	URS / BW URS	3.25–3.49	2.03–2.55	1.61–2.78
	URS / 2HT	1.14–1.26	0.89–1.04	0.88
	Cauda / BW cauda	2.89–3.13	1.57–2.29	1.67–1.80
	Cephalic setae / Ant. III BD	1.51–2.02	1.48–2.04	1.20–1.64
	Setae on tergite I / Ant. III BD	1.10–1.16	1.56–2.19	0.77–0.78
	Setae on tergite VIII / Ant. III BD	2.60–2.73	1.70–2.47	1.33–1.45
	Setae on ANT. III / ANT. III BD	0.76–0.83	0.80–1.32	0.42–0.66
	Setae on hind tibia / MW hind tibia	1.10–1.24	0.98–1.55	0.93–1.06
	SIPH / Body	0.25–0.28	0.12–0.14	0.17–0.18
	SIPH / Cauda	1.97–1.99	1.22–1.48	1.83–1.96
	SIPH / Ant. III	0.79–0.80	0.47–0.58	0.55–0.60
	SIPH / BW SIPH	8.62–10.10	4.64–6.29	7.04–8.62
	SIPH / SW SIPH	5.80–6.22	5.54–6.55	4.28–4.84
	SIPH / DW SIPH	13.69–14.56	6.77–8.55	8.73–9.31

In this paper, the following abbreviations are used: **Ant. I, II, III, IV, V, VIb**: antennal segments I, II, III, IV, V, and the base of antennal segment VI, respectively; **PT**: processus terminalis; **Ant. III BD**: basal diameter of antennal segment III; **URS**: ultimate rostral segment; **BW URS**: basal width of ultimate rostral segment; **MW**: hind tibia: mid-width of hind tibia; **2HT**: second hind tarsal segment; **SIPH**: siphunculus; **BW SIPH**: basal width of siphunculus; **DW SIPH**: distal width of siphunculus; **MW SIPH**: the middle width of basal cylindrical part of siphunculus; **SW SIPH**: the most swollen width of siphunculus; **BW cauda**: basal width of cauda; **Setae on Ant. III**: the longest setae on antennal segment III; **Setae on hind tibia**: the longest setae on hind tibia; **Setae on tergite I**: the longest marginal setae on abdominal tergite I; **Setae on tergite VIII**: the longest spinal setae on abdominal tergite VIII.

DNA barcodes of COI were obtained from Chinese specimens, and the voucher details and sequences were deposited in GenBank (Table 2). Total genomic DNA was extracted from a single apterous aphid, preserved in 95% ethanol, using DNeasy Blood & Tissue Kit (Qiagen, Hilden, Germany). The standard DNA barcode gene of aphids was used and amplified with primers LepF and LepR (Foottit et al. 2008). The methods of PCR thermal regime followed those of Chen et al. (2015). Sequences were assembled by SeqMan II (DNAStar, Inc., Madison, WI, USA) with inspection and manual editing, and then were examined using BLAST to confirm that the sequences were highly similar to other aphid sequences. Multiple alignments were performed with Clustal W (Thompson et al. 1994) and then verified manually. Pairwise genetic distances for the COІ gene were estimated using MEGX (Kumar et al. 2018) under Kimura’s two-parameter (K2P) model (Kimura 1980).

**Table 2.** Voucher and GenBank accession numbers for aphid samples examined in this study.

Species	Voucher number	Collection locality	Host plant	COI
*Indomasonaphis anaphalidis*	52804	China: Xizang	* Anaphalis *	PV567756
*Indomasonaphis anaphalidis*	52782	China: Xizang	* Polygonum *	PV567755
*Indomasonaphis polygoni* sp. nov.	25915	China: Xizang	* Polygonum *	PV567752
*Indomasonaphis polygoni* sp. nov.	25933	China: Xizang	* Polygonum *	PV567753
*Indomasonaphis polygoni* sp. nov.	52759	China: Xizang	* Polygonum *	PV567754
*Indomasonaphis polygoni* sp. nov.	52807	China: Xizang	* Polygonum *	PV567757

The holotype and paratypes of the new species and other specimens examined are deposited in the National Animal Collection Resource Center (NACRC), Institute of Zoology, Chinese Academy of Sciences, Beijing, China.

## ﻿Taxonomy

### ﻿ Indomasonaphis

Taxon classificationAnimaliaHemipteraAphididae

Verma, 1971

95C0DF85-3336-5983-9025-9254F5EBC4E3

Indomasonaphis Verma, 1971: 97. Type species: Indomasonaphis indicum Verma, 1971 (= Masonaphis anaphalidis Basu, 1964), by original designation. Type locality: Shimla, Himachal Pradesh, India.

#### Generic diagnosis.

*Indomasonaphis* species can be recognized by the following combination of characters: body large and elongated oval; body dorsum smooth with sparse long, thick, and blunt or capitate setae; frons concave, median frontal tubercle distinct and low-rounded, antennal tubercles developed protuberate and diverging; antennae 6-segmented, Ant. III mostly without secondary rhinaria in apterae, except in *I. polygoni* sp. nov. with 22–41 secondary rhinaria distributed on the basal half; in alate, Ant. III with 60–130 round secondary rhinaria distributed throughout the segment, Ant. IV with 4–30 secondary rhinaria; PT at least 4.00 times of Ant. VIb; legs long, and with numerous long and pointed setae; 2HT with spinulose imbrications; SIPH long and clavate, cylindrical at basal 1/3, distinctly expanded at distal 2/3, and then gradually attenuated to apex; apical part of SIPH with several transverse rows of imbrications, sometimes coalescing into reticulations, and with a developed flange; cauda wide and long conical, with numerous setae; first tarsal chaetotaxy: 5, 5, 5, or 3, 3, 3.

#### Taxonomic comments.

The genus resembles *Illinoia* Wilson, 1910 in the clavate SIPH and conical cauda, but differs from it as follows: (1) SIPH long, clavate, cylindrical at basal 1/3 and distinctly expanded at distal 2/3 (*Illinoia*: SIPH long, cylindrical, sometimes weakly to moderately swollen at distal 1/3); (2) dorsal setae long, thick, and blunt (*Illinoia*: dorsal setae short and blunt); (3) Ant. III mostly without secondary rhinaria in apterae (*Illinoia*: Ant. III with at least two secondary rhinaria in apterae); and (4) cauda wide and long conical, with numerous long and pointed setae (*Illinoia*: cauda elongate conical, bearing 6–10 short and pointed setae).

The genus is also similar to *Chaetomyzus* Ghosh & Raychaudhuri, 1962 in the clavate SIPH and spinulose 2HT, but differs from it as follows: (1) median frontal tubercle slightly swollen, antennal tubercles developed protuberate and diverging (*Chaetomyzus*: median frontal tubercle slightly swollen, antennal tubercles distinct but low-rounded and diverging); (2) body dorsum smooth, without tubercles (*Chaetomyzus*: body dorsum with paired processes); (3) PT at least 4.00 × of Ant. VIb (*Chaetomyzus*: PT at most 2.00 × of Ant. VIb); and (4) cauda wide and long conical, with numerous setae (*Chaetomyzus*: cauda conical, with few setae).

The genus also resembles *Liosomaphis* Walker, 1868 in the clavate SIPH, but differs from it as follows: (1) frons concave, median frontal tubercle slightly swollen, antennal tubercles developed protuberate and diverging (*Liosomaphis*: frons shallow W-shaped, antennal tubercles low-rounded, median frontal tubercle distinctly protuberate, higher than antennal tubercles); (2) antennae as long as body length, PT at least 4.00 × of Ant. VIb (*Liosomaphis*: antennae ~0.50 × of body length, PT ~1.50 × of Ant. VIb); (3) dorsal setae long, thick, and blunt (*Liosomaphis*: dorsal setae short and point).

#### Distribution.

China, India, Pakistan.

### ﻿ Indomasonaphis anaphalidis

Taxon classificationAnimaliaHemipteraAphididae

(Basu, 1964)

3AE23414-E327-52CA-9D51-33C898D80AF2

**Figure 1.**
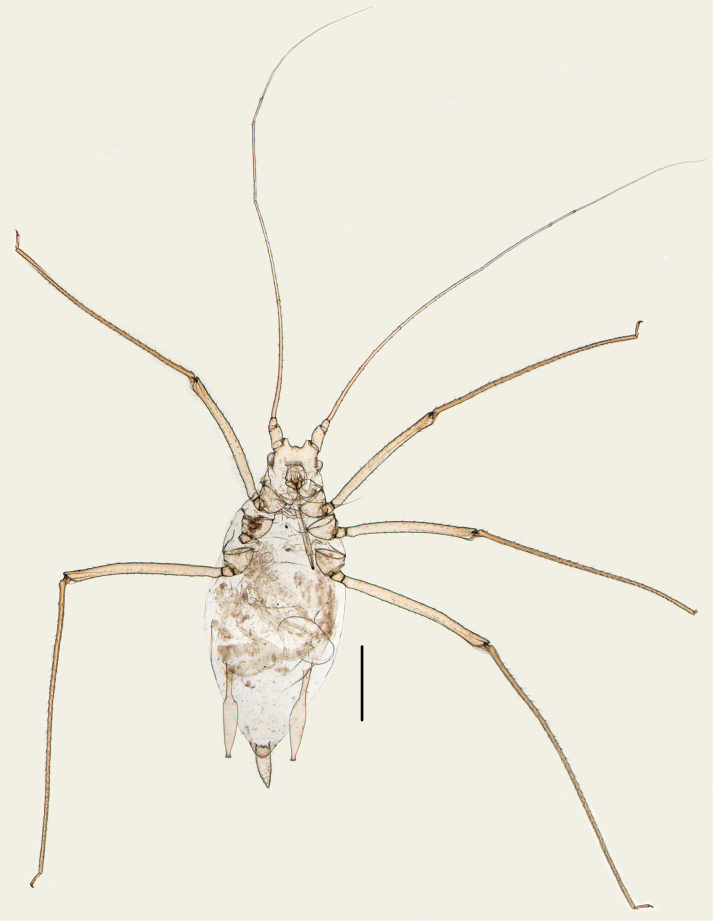
Specimen No. 45445-1-1: habitus of apterous viviparous female of *Indomasonaphis anaphalidis* (Basu). Scale bar: 1.00 mm.

**Figure 2.**
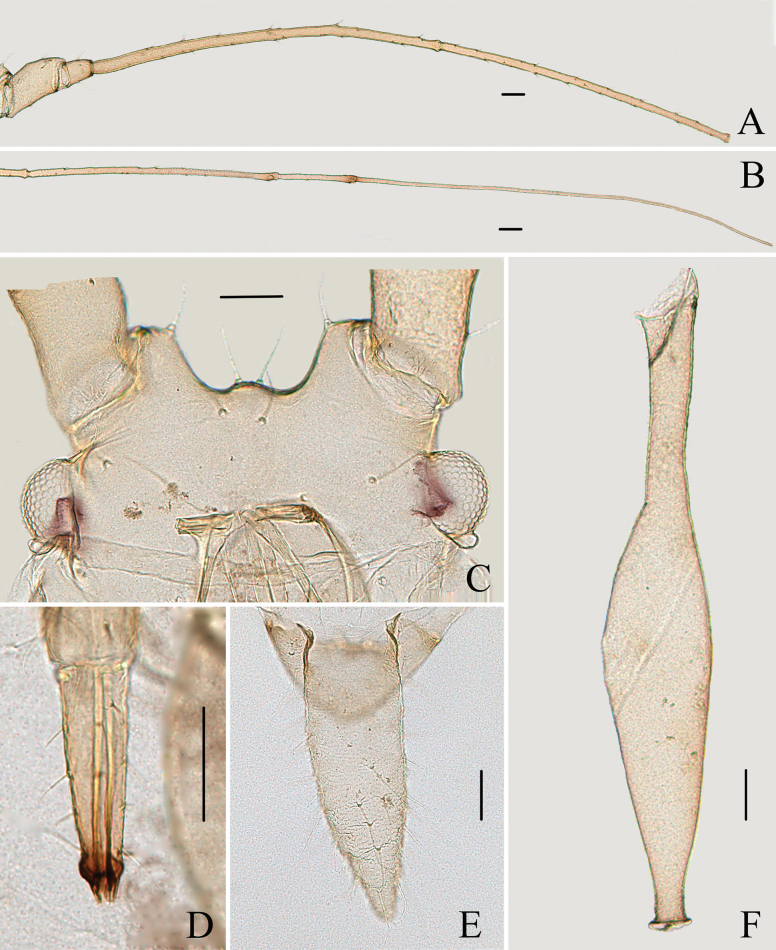
Specimen No. 45445-1-1: apterous viviparous female of *Indomasonaphis anaphalidis* (Basu): A. Antennal segments I–IV; B. Antennal segments V–VI; C. Dorsal view of head; D. Ultimate rostral segment; E. Cauda; F. Siphunculus. Scale bars: 0.10 mm.

**Figure 3.**
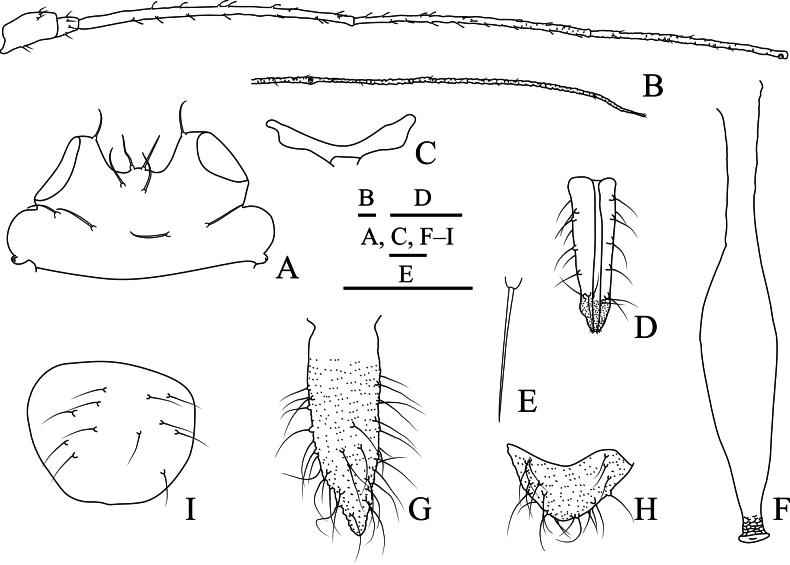
Specimen No. 45445-1-1: apterous viviparous female of *Indomasonaphis anaphalidis* (Basu): A. Dorsal view of head; B. Antenna; C. Mesosternal furca; D. Ultimate rostral segment; E. Marginal seta of abdominal tergite III; F. Siphunculus; G. Cauda; H. Anal plate; I. Genital plate. Scale bars: 0.10 mm.

Figs 1, 2, 3, 12, Table 1

#### Specimens examined.

**China: Xizang.** • One apterous viviparous female, Cuona County, 19.VIII.2019, No. 46078-1-1, on *Senecio* sp., coll. T. T. Xu; • one apterous viviparous female, 13.VII.2021, No. 51872-1-1, on *Senecio* sp., coll. Y. Xu; • one apterous viviparous female, Yadong County, 9.VII.2022, No. 52804-1-1, on *Anaphalis* sp., coll. Z. X. Li; • one apterous viviparous female (COI GenBank accession: PV567756), with the same collection information as No. 52804; • one apterous viviparous female, 7.VII.2022, No. 52782-1-1, on *Polygonum* sp., coll. Z. X. Li; • one apterous viviparous female (COI GenBank accession: PV567755), with the same collection information as No. 52782.

#### Diagnosis.

URS long wide-shaped, 3.25–3.49 × of basal width, 1.14–1.26 × of 2HT, with 12–15 accessory setae (Figs 2D, 3D); Ant. III–IV in alatae with 125–130, 9–12 round, protuberant secondary rhinaria, respectively; SIPH long clavate (Figs 2F, 3F), thin and cylindrical at basal 1/3, then distinctly expanded at distal 2/3, and then gradually attenuated to apex, apical part of SIPH with 5–8 rows reticulations, 0.25–0.28 × of body length; the SW SIPH 1.39–1.74 × of BW SIPH; cauda long conical (Figs 2E, 3G), 2.89–3.13 × of BW cauda, with 41–43 setae; first tarsal chaetotaxy: 5, 5, 5.

#### Biology.

The species alternates between *Rhododendron* (primary hosts) and Asteraceae (secondary hosts, including *Anaphalis*, *Artemisia*, *Gerbera*, *Inula*, and *Senecio*), but it has also been recorded on some atypical host plants (*Eurya japonica*, *Morus alba*, *Oxalis corniculata*) (Verma 1971; Chakrabarti et al. 1983; Favert and Aphid Taxon Community 2025). The aphid feeds on the underside of leaves of *Senecio* sp. and *Anaphalis* sp. without causing noticeable damage (Fig. 12), and has additionally been observed infesting *Polygonum* sp. in China.

#### Distribution.

India: Himachal Pradesh (Verma 1971; Chakrabarti et al. 1983), Uttarakhand (Chakrabarti et al. 1983), Uttar Pradesh (Chakrabarti et al. 1983), West Bengal (Basu 1964); Pakistan: Murree (Muhammad et al. 2024). This species is recorded here for the first time from China, in Xizang Autonomous Region.

### ﻿ Indomasonaphis polygoni

Taxon classificationAnimaliaHemipteraAphididae

Qiao & Xu sp. nov.

1406578A-DF70-5E8E-9667-92219AAED55D

https://zoobank.org/002F4526-7517-4D89-BB8D-4DC03BE1526A

**Figure 4.**
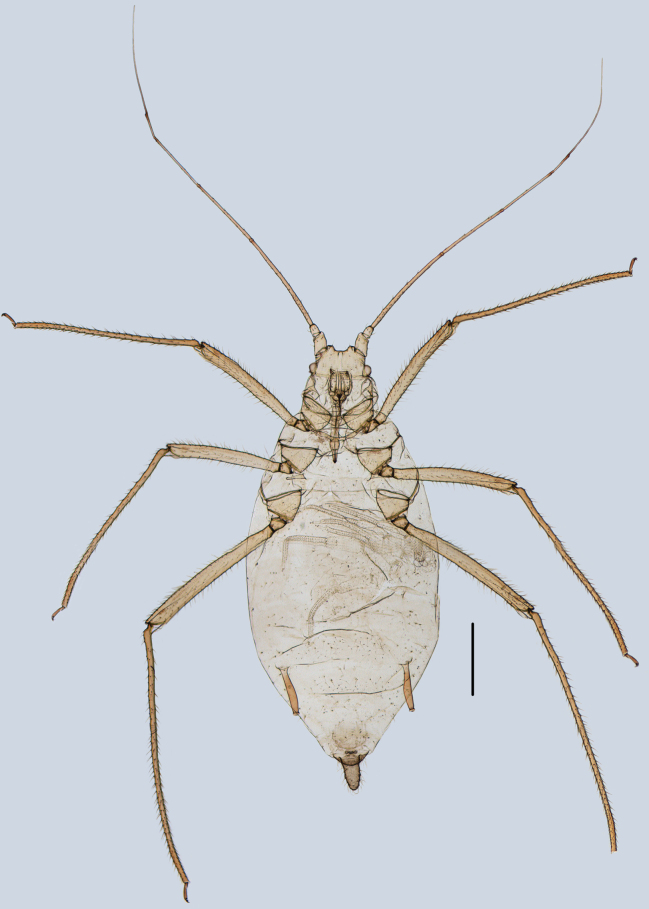
Specimen No. 51934-1-1: habitus of apterous viviparous female of *Indomasonaphis polygoni* Qiao & Xu, sp. nov. Scale bar: 1.00 mm.

**Figure 5.**
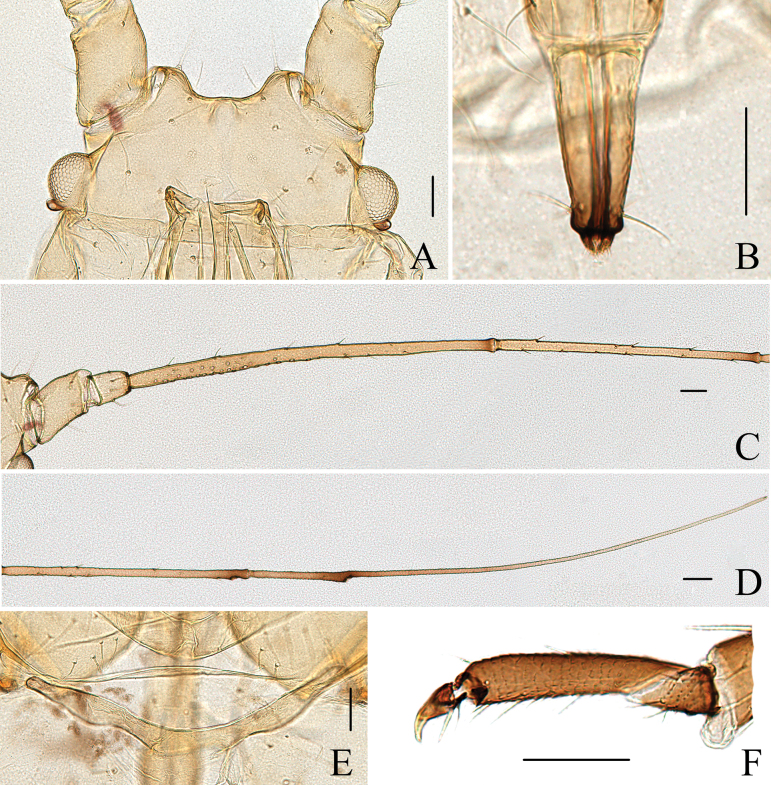
Specimen No. 51934-1-1: apterous viviparous female of *Indomasonaphis polygoni* Qiao & Xu, sp. nov.: A. Dorsal view of head; B. Ultimate rostral segment; C. Antennal segments I–IV; D. Antennal segments V–VI; E. Mesosternal furca; F. Hind tarsus. Scale bars: 0.10 mm.

**Figure 6.**
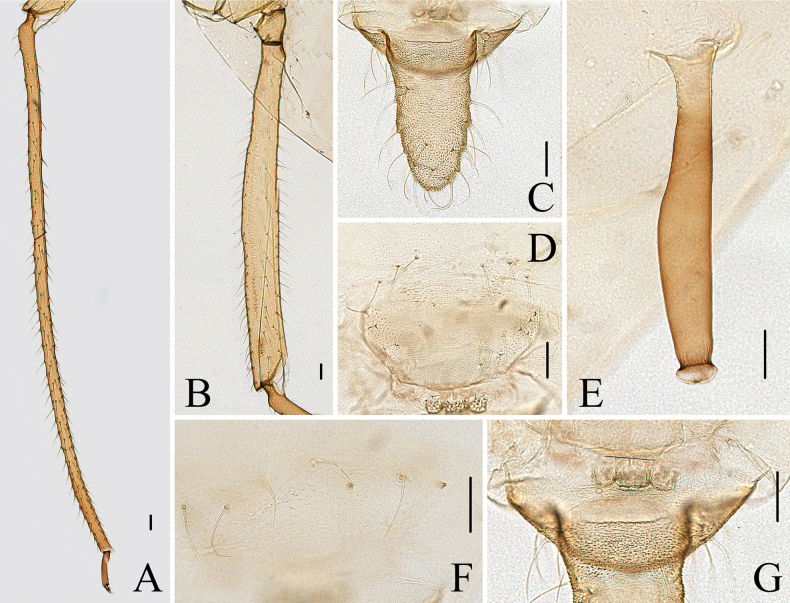
Specimen No. 51934-1-1: apterous viviparous female of *Indomasonaphis polygoni* Qiao & Xu, sp. nov.: A. Hind tibia; B. Hind femur; C. Cauda; D. Genital plate; E. Siphunculus; F. Setae on abdominal tergite VIII; G. Anal plate. Scale bars: 0.10 mm.

**Figure 7.**
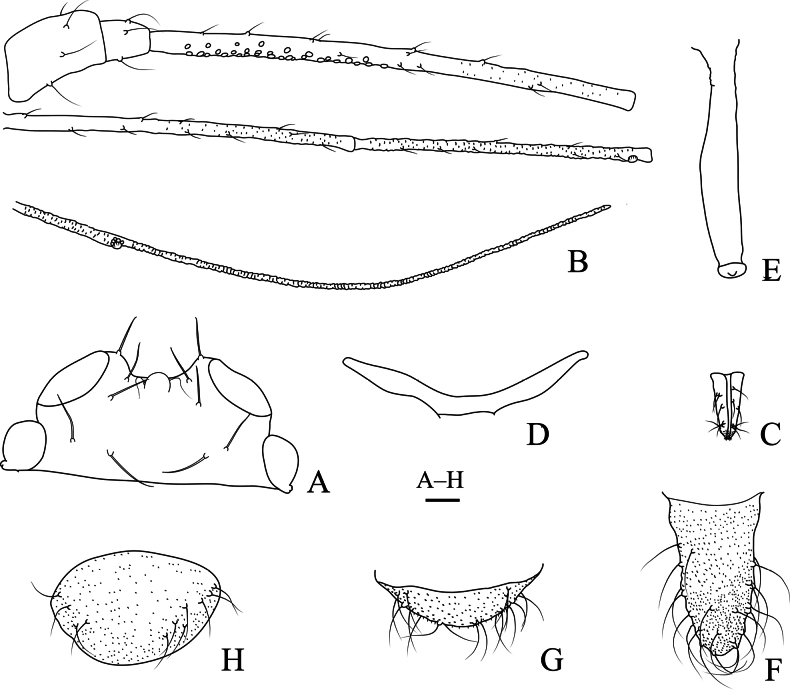
Specimen No. 51934-1-1: apterous viviparous female of *Indomasonaphis polygoni* Qiao & Xu, sp. nov.: A. Dorsal view of head; B. Antenna; C. Ultimate rostral segment; D. Mesosternal furca; E. Siphunculus; F. Cauda; G. Anal plate; H. Genital plate. Scale bars: 0.10 mm.

Figs 4, 5, 6, 7, 13A–D, Table 1

#### Type material.

***Holotype*: China: Xizang**: Yadong County, • one apterous viviparous female, 20.VII.2021, No. 51934-1-1, on *Polygonum* sp., coll. Y. Xu. ***Paratypes* (11): China: Xizang**: Yadong County, • one apterous viviparous female, 16.VIII.2020, No. 25915-1-1, on *Polygonum* sp., coll. Y. Wang; • one apterous viviparous female (COI GenBank accession: PV567752), with the same collection information as No. 25915; • one apterous viviparous female, 17.VIII.2020, No. 25933-1-1, on *Polygonum* sp., coll. Q. H. Liu; • one apterous viviparous female (COI GenBank accession: PV567753), with the same collection information as No. 25933; • one apterous viviparous female, 17.VII.2014, No. 32677-1-1, on *Polygonum* sp., coll. J. Chen and X. C. Zhu; • two apterous viviparous females, 20.VII.2021, No. 51931-1-1, 51931-2-1, on *Polygonum* sp., coll. Y. Xu; • one apterous viviparous female, 7.VII.2022, No. 52759-1-1, on *Polygonum* sp., coll. Z. X. Li; • one apterous viviparous female (COI GenBank accession: PV567754), with the same collection information as No. 52759; • one nymph (COI GenBank accession: PV567757), 7.VII.2022, No. 52807-1-1, on *Polygonum* sp., coll. Z. X. Li; • one apterous viviparous female, Linzhi County (Lulang Town), 3.VIII.2014, No. 32890-1-1, on *Polygonum* sp., coll. J. Chen and X. C. Zhu; one apterous viviparous female, Milin County, 30.VIII.2020, No. 49106-1-1, on *Polygonum* sp., coll. Y. Xu.

#### Diagnosis.

Body large (Fig. 4), length 5.15–6.37 mm. Ant. III with 22–41 secondary rhinaria in apterae, distributed on basal part (Figs 5C, 7B); SIPH clavate, cylindrical at basal 1/4, then expanded at distal 3/4, and slightly attenuated at apex, smooth, and with developed flange (Figs 6E, 7E), 0.12–0.16 × body length; cauda long and wide conical (Figs 6C, 7F), length 1.57–2.29 × basal width, with 15–29 setae; first tarsal chaetotaxy: 5, 5, 5.

#### Description.

***Apterous viviparous females***: body large, elongated oval (Fig. 4). Green in life, with red compound eyes, intersegmental regions of the dorsum and pleural areas yellowish-green, distal part of appendages pale brown (Fig. 13D). For morphometric data see Table 1.

***Mounted specimens*.** Body dorsum pale, smooth, without sclerites; Ant. I and II pale, III–VI pale brown with intersegmental areas brown; apex of rostrum brown; distal part of tibiae and tarsi brown, remaining parts of leg pale brown; SIPH brown, but pale near base; cauda and anal plate pale brown (Fig. 4). Dorsal setae long, thick, and blunt at apexes; ventral setae long and pointed, as long as dorsal setae.

***Head*.** Frons concave, median frontal tubercle moderately swollen, low-rounded, antennal tubercles developed, distinctly protuberate and diverging, and each with one seta at apex (Figs 5A, 7A). Head with one pair of cephalic setae, two pairs of dorsal setae between antennae arranged longitudinally, two pairs of dorsal setae between compound eyes arranged transversely. Antennae 6-segmented (Figs 5C, D, 7B), Ant. I–III smooth, IV–VI imbricated; antennal setae short and blunted, segments I–VI each with 4–6, 4, 11–14, 9–15, 4–7, 2–4+2–6 setae, respectively, processus terminalis with three apical setae. Primary rhinaria ciliated. Ant. III with 22–41 secondary rhinaria, distributed on the basal half (Figs 5C, 7B). Rostral apex reaching mid-coxae, URS wedge-shaped (Figs 5B, 7C), with three pairs of primary setae and 6–10 accessory setae.

***Thorax*.** Thoracic nota smooth. Pronotum with four spinal setae, one pair of pleural setae, and one pair of marginal setae; mesonotum and metanotum each bearing 4–14 spino-pleural setae and two pairs of marginal setae. Mesosternal furca with short stem (Figs 5E, 7D). Legs long. Femora with oval sculpturing on dorsal apices; distal parts of tibiae imbricated. Setae on legs long and pointed, moderately stout, densely distributed over entire segments (Fig. 6A, B). First tarsal chaetotaxy: 5, 5, 5. Second tarsal segments with spinulose imbrications (Fig. 5F).

***Abdomen*.** Abdominal tergites I–VI each with 5–16 spino-pleural setae, 2–7 pairs of marginal setae; abdominal tergites VIII with 5–12 setae (Fig. 6F). Spiracles circular, closed; spiracular plates slightly swollen, pale brown. SIPH clavate, broad at base, basal 1/4 cylindrical, then expanded at distal 3/4, and slightly attenuated at apex, smooth without imbrications, and with developed flange (Figs 6E, 7E). Cauda long and wide conical, finely spinulose (Figs 6C, 7F), with 15–29 densely arranged long setae. Anal plate semi-circular, with spinulose striae and bearing 16–28 setae (Figs 6G, 7G). Genital plate broadly oval (Fig. 7H), with densely spinulose striae, and 4–12 anterior setae and 11–23 setae along the posterior margin.

#### Etymology.

The species name is based on the generic epithet of its host plant *Polygonum* (Polygonaceae).

#### Taxonomic discussion.

The species resembles *I. anaphalidis*, but differs from it as follows: (1) the species feeds on *Polygonum* sp. (*I. anaphalidis*: the primary host plants are *Rhododendron* sp., and the secondary host plants mainly belong to the family Asteraceae); (2) Ant. III with 22–41 secondary rhinaria in apterae, distributed on basal part (*I. anaphalidis*: Ant. III without secondary rhinaria); (3) SIPH clavate, 0.12–0.16 × body length, cylindrical at basal 1/4, then expanded at distal 3/4, the SW SIPH 1.39–1.89 × MW SIPH, and then slightly attenuated at apex, smooth without imbrications, and with developed flange (*I. anaphalidis*: SIPH long clavate, 0.25–0.28 × body length, cylindrical at basal 1/3, then distinctly expanded at distal 2/3, the SW SIPH 2.79–2.81 × MW SIPH, and then gradually attenuated to apex, apical part of SIPH with 5–8 rows reticulations); and (4) cauda long and wide conical, length 1.57–2.29 × basal width, with 15–29 setae (*I. anaphalidis*: cauda elongated conical, length 2.89–3.13 × basal width, with 41–43 setae).

The species can be distinguished from *I. rumicis* by follows: (1) Ant. III with 22–41 secondary rhinaria in apterae, distributed on basal part (*I. rumicis*: Ant. III without secondary rhinaria); (2) first tarsal chaetotaxy: 5, 5, 5 (*I. rumicis*: first tarsal chaetotaxy: 3, 3, 3); (3) dorsal setae long, thick, and blunt at apex (*I. rumicis*: dorsal setae long, thick, and capitate at apex); (4) SIPH clavate, 0.12–0.16 × body length, cylindrical at basal 1/4, then slightly expanded at distal 3/4, the SW SIPH 1.39–1.89 × MW SIPH, and then slightly attenuated at apex, smooth without imbrications (*I. rumicis*: SIPH clavate, 0.17–0.18 × body length, cylindrical at basal 1/2, then distinctly expanded at distal 1/2, the SW SIPH 2.32–2.91 × MW SIPH, and then gradually attenuated to apex, apical part of SIPH with 2–4 rows of imbrications).

#### Biology.

The species feeds on the underside of leaves of *Polygonum* sp. without causing noticeable damage (Fig. 13A–D).

#### Distribution.

China (Xizang: Linzhi, Yadong).

### ﻿ Indomasonaphis rumicis

Taxon classificationAnimaliaHemipteraAphididae

(Chakrabarti & Raychaudhuri, 1975)

F0AFA580-0733-5938-BE77-9E75A1C6C199

**Figure 8.**
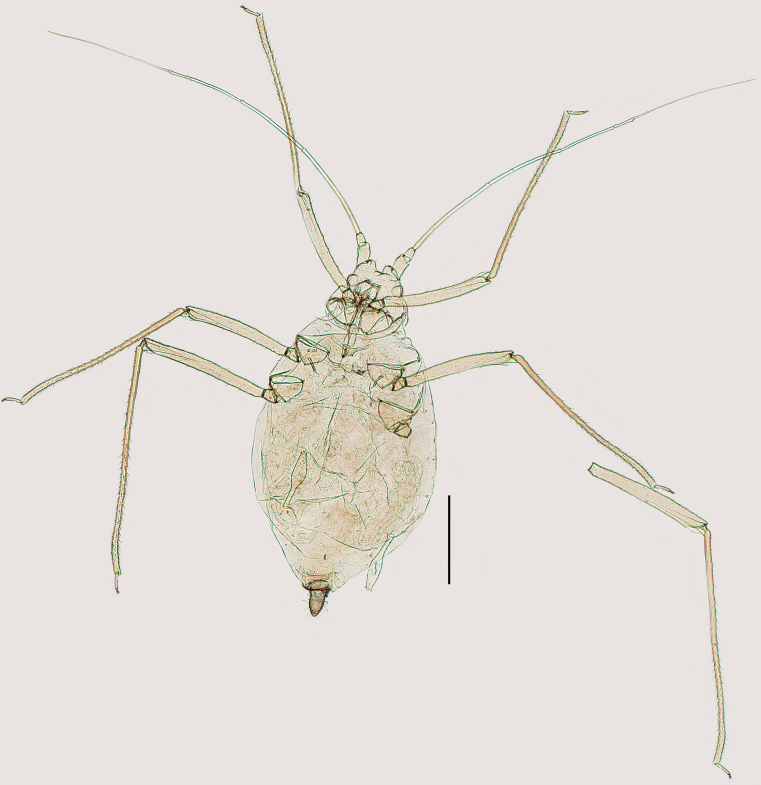
Specimen No. 25927-1-1: habitus of apterous viviparous female of *Indomasonaphis rumicis* (Chakrabarti & Raychaudhuri). Scale bar: 1.00 mm.

**Figure 9.**
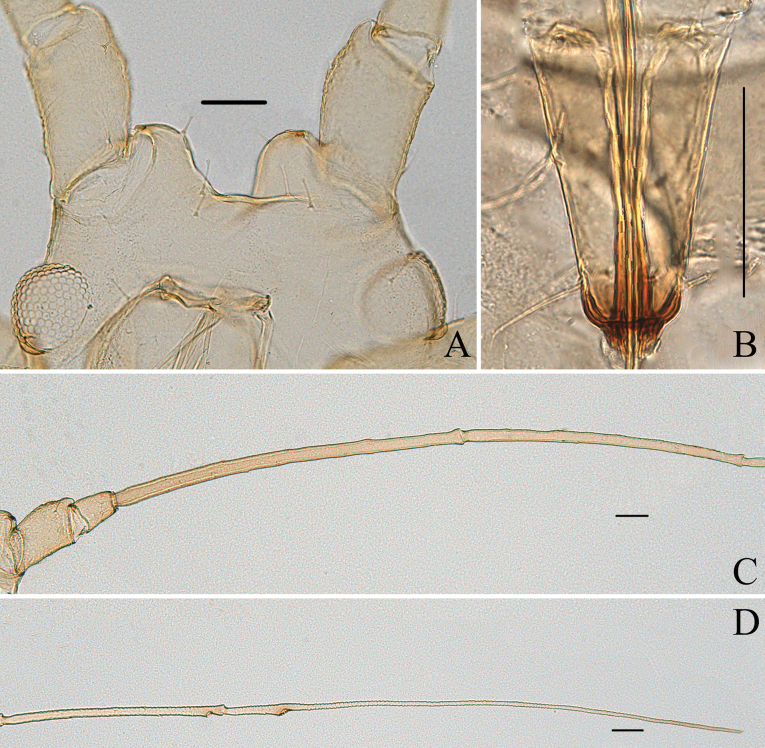
Specimen No. 25927-1-1: apterous viviparous female of *Indomasonaphis rumicis* (Chakrabarti & Raychaudhuri): A. Dorsal view of head; B. Ultimate rostral segment; C. Antennal segments I–IV; D. Antennal segments V–VI. Scale bars: 0.10 mm.

**Figure 10.**
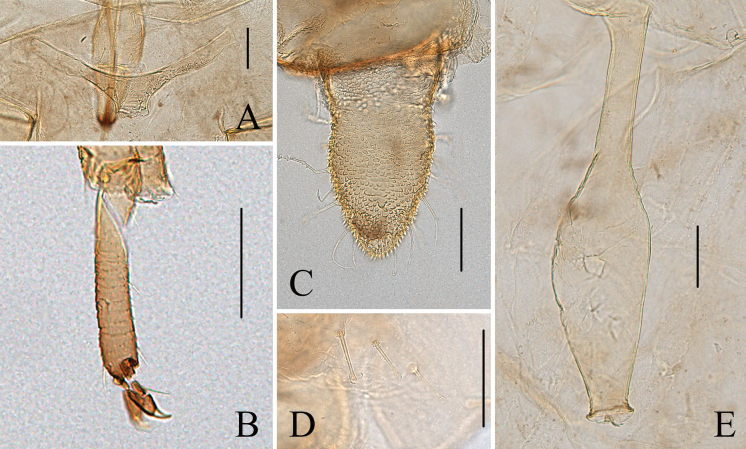
Specimen No. 25927-1-1: apterous viviparous female of *Indomasonaphis rumicis* (Chakrabarti & Raychaudhuri): A. Mesosternal furca; B. Hind tarsus; C. Cauda; D. Setae on abdominal tergite VIII; E. Siphunculus. Scale bars: 0.10 mm.

**Figure 11.**
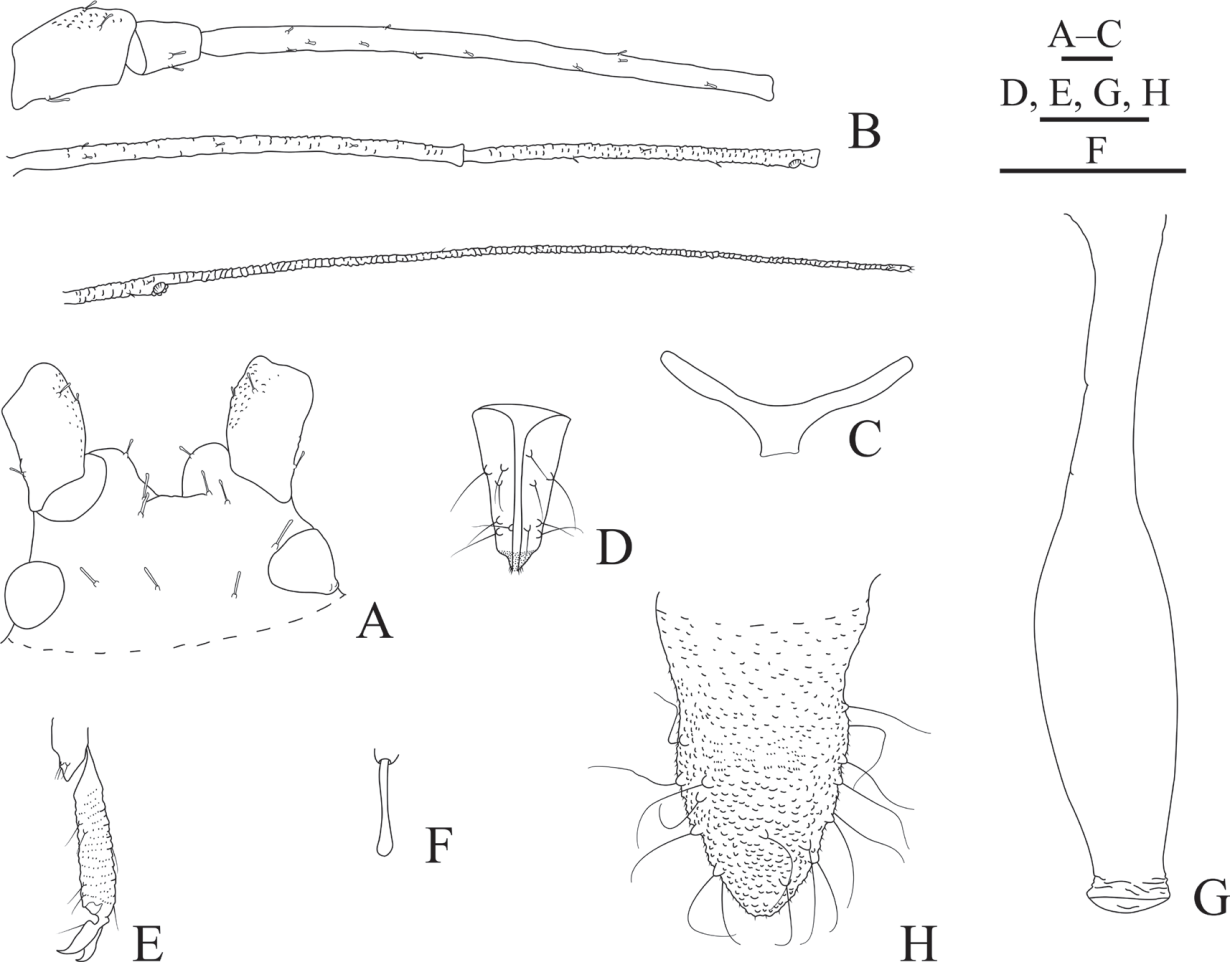
Specimen No. 25927-1-1: apterous viviparous female of *Indomasonaphis rumicis* (Chakrabarti & Raychaudhuri): A. Dorsal view of head; B. Antenna; C. Mesosternal furca; D. Ultimate rostral segment; E. Hind tarsus; F. Marginal seta of abdominal tergite I; G. Siphunculus; H. Cauda. Scale bars: 0.10 mm.

Figs 8, 9, 10, 11, 13E, F, Table 1

#### Specimens examined.

**China: Xizang.** • One apterous viviparous female, Yadong County, 17.VIII.2010, No. 25927-1-1, on *Rumex* sp., coll. Y. Wang; • one apterous viviparous female, Zhangmu County, 3.VIII.2019, No. 46042-1-1, on *Rumex* sp., coll. T. T. Xu.

#### Diagnosis.

URS wedge-shaped (Figs 9B, 11D), 1.67–2.78 × basal width, 0.88 × 2HT, with 4–6 accessory setae; Ant. III–IV in alatae with 63–67, 4–6 secondary rhinaria, respectively; SIPH long clavate (Figs 10E, 11G); cylindrical at basal 1/2, then distinctly expanded at distal 1/2, and then gradually attenuated to apex, apical part of SIPH with 2–4 rows of imbrications, 0.17–0.18 × body length; cauda wide conical (Figs 10C, 11H), 1.67–1.80 × BW cauda, with 15–17 setae; first tarsal chaetotaxy: 3, 3, 3.

#### Biology.

The species feeds on the underside of leaves of *Rumex* sp. without causing noticeable damage (Fig. 13E, F), and also has been recorded on *Oxyria digyna* and *Gerbera* sp. in India (Chakrabarti and Raychaudhuri 1975).

**Figure 12.**
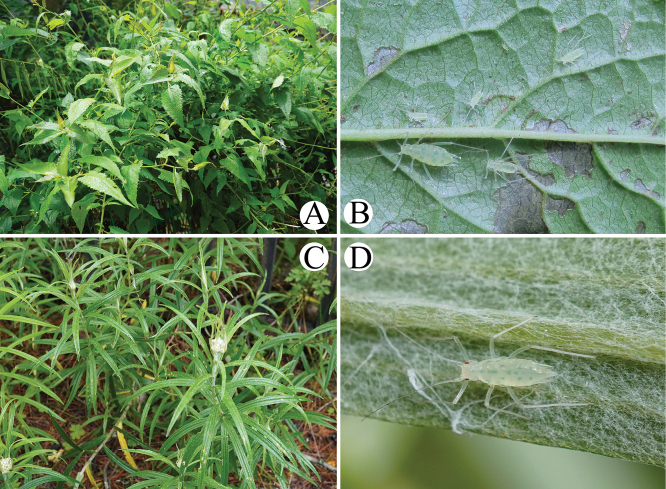
*Indomasonaphis anaphalidis* (Basu): A. The host plant *Senecio* sp. of *I. anaphalidis*; B. Apterae of *I. anaphalidis* feeding on the underside of leaves of *Senecio* sp.; C. The host plant *Anaphalis* sp. of *I. anaphalidis*; D. Apterae of *I. anaphalidis* feeding on the underside of leaves of *Anaphalis* sp.

**Figure 13.**
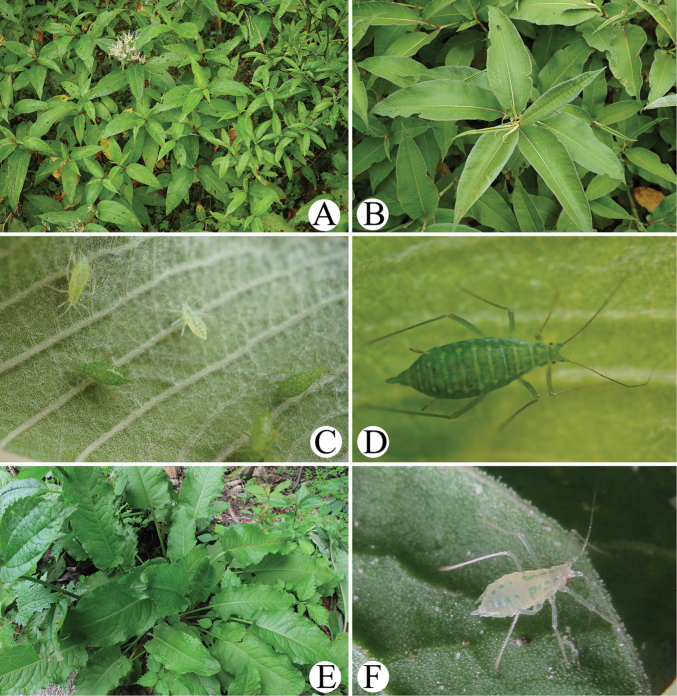
*Indomasonaphis polygoni* Qiao & Xu, sp. nov.: A, B. The host plant *Polygonum* sp. of *I. polygoni* sp. nov.; C. Apterae of *I. polygoni* sp. nov. feeding on the underside of leaves of *Polygonum* sp.; D. One aptera of *I. polygoni* sp. nov.; *Indomasonaphis rumicis* (Chakrabarti & Raychaudhuri); E. The host plant *Rumex* sp. of *I. rumicis* sp. nov.; F. One aptera of *I. rumicis* feeding on the underside of leaves of *Rumex* sp.

#### Distribution.

India: Uttar Pradesh (Chakrabarti and Raychaudhuri 1975).

This species is recorded here for the first time from China, in Xizang Autonomous Region.

### ﻿Key to species of *Indomasonaphis* Verma, 1971 in China

**Table:** 

1	URS long wedge-shaped, 1.14–1.26 × 2HT, with 12–15 accessory setae; cauda long, conical, length 2.89–3.13 × basal width, with 41–43 setae; SIPH with 5–8 rows of reticulations at distal part	***I. anaphalidis***
–	URS wedge-shaped, less than 1.00 × 2HT, with 4–10 accessory setae; cauda wide conical, length ~2.00 × basal width, with 15–29 setae; SIPH with several rows of imbrications or smooth at distal part	**2**
2	Ant. III without secondary rhinaria in apterae; PT ~7.00 × Ant.VIb; cauda 1.67–1.80 × BW cauda; SIPH cylindrical at basal 1/2, then distinctly expanded at distal 1/2, with 2–4 rows of imbrication at distal part	***I. rumicis***
–	Ant. III with 22–41 secondary rhinaria in apterae; PT 3.96–4.71 × Ant.VIb; cauda 1.57–2.29 × BW cauda; SIPH cylindrical at basal 1/4, then slightly expanded at distal 3/4, smooth at distal part	***I. polygoni* sp. nov.**

### ﻿Updated key to aphids feeding on *Polygonum* (from Favret and Aphid Taxon Community 2025)

**Table:** 

43	SIPH moderately to markedly clavate, smooth-surfaced	**44a**
–	SIPH not clavate; tapering/cylindrical or very slightly swollen, imbricated or thick and scabrous	**45**
44a	Abdominal tergites I and VII always with small tubercles; appendages mainly dark; dorsal cuticle with a pattern of spinules arranged in polygons	***Rhopalosiphum nymphaeae***
–	Abdominal tergites I and VII without small tubercles; appendages pale; dorsal cuticle smooth, not spinulose	**44b**
44b	Median frontal tubercle distinctly protuberant and rectangular, higher than antennal tubercles; abdominal tergites VIII, or VII and VIII, with a median tubercular process bearing a pair of short blunt setae	***Tricaudatus polygoni***
–	Median frontal tubercle slightly swollen; antennal tubercles developed protuberate and diverging, distinctly higher than median frontal tubercles; abdominal tergites VII and VIII without median tubercular processes	***Indomasonaphis polygoni* sp. nov.**

### ﻿DNA barcoding

The DNA barcodes of two species in the genus were acquired for the first time, including *I. anaphalidis* and *I. polygoni* sp. nov. The interspecific genetic distances between the two species are 11.51–11.88%. Due to limited sample availability, DNA barcode sequences could not be obtained for *I. rumicis*. Based on DNA barcodes, the validity of new species is supported. Additional specimen collections are required to further support the taxonomic delineation within this genus.

## ﻿Discussion

Species of *Indomasonaphis* are distributed in the southern Himalayan region, with most specimens collected from areas surrounding the Himalayan Mountains. Therefore, its evolution may be related to the uplift of the Qinghai-Tibet Plateau. Each species of this genus possesses its own distinctive morphological features: the type species, *I. anaphalidis*, exhibits long clavate SIPH with 5–8 rows of reticulations at the distal part, and conspicuously hairy cauda; *I. chakrabartii* has long, capitate setae on tuberculate bases, and long cylindrical SIPH with 3–5 rows of reticulations at the distal part; *I. inulae* is with long thin URS, 2.00 × 2HT, long cylindrical SIPH, and capitate setae on tuberculate bases; *I. polygoni* sp. nov. has clavate and smooth SIPH, and Ant. III with 22–41 secondary rhinaria in apterae; *I. rumicis* has long clavate SIPH with 5–8 rows of reticulations at the distal part, and the first tarsal chaetotaxy is 3, 3, 3.

The morphological diversity within this genus raises doubts about its monophyly. Therefore, additional and reliable molecular data will be necessary to assess its taxonomic validity. Also, the biological knowledge of this genus remains incomplete and requires further investigation. Although the primary host plants of some species are *Rhododendron* and secondary hosts are Asteraceae, the host relationships of other congeneric species remain unclear. Therefore, expanded sampling efforts across potential host plants are essential to clarify biological information, obtain critical molecular markers, and reassess diagnostic morphological characters through integrated analyses.

## Supplementary Material

XML Treatment for Indomasonaphis

XML Treatment for Indomasonaphis anaphalidis

XML Treatment for Indomasonaphis polygoni

XML Treatment for Indomasonaphis rumicis

## ﻿Additional information

### Use of AI

No use of AI was reported.

### Author ORCIDs

Shokhruz Nazarov https://orcid.org/0000-0002-9256-7885

Ying Xu https://orcid.org/0000-0002-8950-7718

Li-Yun Jiang https://orcid.org/0000-0002-2527-9613

Ge-Xia Qiao https://orcid.org/0000-0002-7300-6812

### Data availability

All of the data that support the findings of this study are available in the main text.
